# Chemical profiling of mycosporine‐like amino acids in twenty‐three red algal species

**DOI:** 10.1111/jpy.12827

**Published:** 2019-01-31

**Authors:** Maria Orfanoudaki, Anja Hartmann, Ulf Karsten, Markus Ganzera

**Affiliations:** ^1^ Institute of Pharmacy, Pharmacognosy University of Innsbruck Innrain 80‐82 Innsbruck 6020 Austria; ^2^ Institute of Biological Sciences Applied Ecology & Phycology University of Rostock Albert‐Einstein‐Str. 3 Rostock 18059 Germany

**Keywords:** isolation, LC‐MS, mycosporine‐like amino acids, NMR, purification, red algae

## Abstract

Rhodophyta produce a variety of chemically different mycosporine‐like amino acids (MAAs), compounds that are known as some of the strongest ultraviolet (UV) absorbing molecules in nature. Accordingly, they primarily act as photoprotectants against harmful levels of solar ultraviolet radiation in the UV‐A and UV‐B range. In order to get a deeper understanding of the chemical diversity of MAAs in red algae, pure standards of eleven mycosporine‐like amino acids were isolated from three different species (*Agarophyton chilense*,* Pyropia plicata* and *Champia novae‐zelandiae*) using various chromatographic methods. Their structures were confirmed by nuclear magnetic resonance and mass spectrometry. Four out of the eleven MAAs are reported for the first time in algae. In addition, a new high‐performance liquid chromatography method was developed for the separation of all isolated MAAs and successfully applied for the analysis of twenty‐three red algal species of marine origin. All of them contained MAAs, the most abundant compounds were shinorine, palythine, asterina‐330 and porphyra‐334. For some samples, the direct assignment of MAAs based on their UV spectra was not possible; therefore, the target analytes were enriched by a simple concentration step, followed by liquid chromatography‐mass spectrometry analysis of the extracts. This approach enabled a deeper insight into the MAA pattern of red algae, indicating that not only the four dominant ones are synthesized but also many others, which were often described as unknown compounds in previous studies.

**Abbreviations**
D_2_O: deuterated water
FCPC: fast centrifugal partition chromatography
LC‐DAD‐ESI–MS: liquid chromatography ‐ diode array detector ‐ electrospray ionization‐mass spectrometry
LC‐MS: liquid chromatography‐mass spectrometry
MAAs: mycosporine‐like amino acids
NOESY: nuclear overhauser enhancement spectroscopy
TMS: tetramethylsilane

The effects of increased levels of ultraviolet radiation (UVR) reaching the earth's surface have become an important health and ecological concern, mainly as a consequence deriving from ozone‐depleting substances such as chlorofluorocarbons in the atmosphere (Cardozo et al. 2011, Montzka et al. 2018). These compounds were banned by the Montreal Protocol in 1987 already; however, many uncontrolled substances like dichloromethane are still under suspicion to also cause ozone reduction (Hossaini et al. 2017). UVR is strongly affecting many benthic marine organisms in the shallow water zone such as seaweeds (Karsten 2007). Algae have developed various adaptation mechanisms to withstand the harmful effects of UV‐A (315–400 nm) and UV‐B (280–315 nm; Karsten 2007 and references therein), like self‐shading by mat formation (Blindow and Schütte 2007), dissipation of excess energy as heat (Ramanan et al. 2014), development of anti‐oxidative systems involving enzymatic and non‐enzymatic mechanisms (Lee and Shiu 2009, Ramanan et al. 2014), and the accumulation of primary (Hartmann et al. 2015a) or secondary metabolites such as mycosporine‐like amino acids. MAAs are composed of a cyclohexenone or cyclohexenimine chromophore conjugated with the nitrogen substituent of an amino acid or its imino alcohol (Sinha et al. 2007) and characterized by their small molecular weight, high solubility, and polarity (Wada et al. 2015). They are among the strongest UV‐absorbing natural products and their absorption maxima are stretched between 268 and 362 nm depending on their chemical structure. Therefore, MAAs increase the UV‐absorbing capacity of respective organisms (Jansena et al. 1998), and are interesting for cosmetic and pharmaceutical use as active ingredients in cosmeceuticals (Rastogi et al. 2017). Rhodophyta are an ideal source for MAAs because they are known to accumulate the highest concentrations per dry weight and to contain the greatest variety among the different algal divisions, with shinorine, porphyra‐334, palythine, asterina‐330, mycosporine‐glycine, usujirene, palythinol and palythene being the most abundant representatives (Karsten et al. 1998, 2006). Green algae (i.e., Chlorophyta and Streptophyta) on the other hand, comprise a generally lower number of species containing MAAs, but some terrestrial genera (e.g., *Prasiola*,* Klebsormidium*) exhibit comparable or even higher concentrations of specific MAAs compared to Rhodophyta (Kitzing et al. 2014, Hartmann et al. 2016), whereas Phaeophyceae do not contain MAAs in general or exhibit only trace amounts (which might derive from epiphytic algae); furthermore, cyanobacteria have been reported to produce a high variety of MAAs, many of them remain chemically unknown (Carreto and Carignan 2011). Several studies revealed geographic, seasonal and bathymetric trends, showing that red algae from tropical zones accumulate higher amounts of MAAs than those in temperate regions. In general, their concentrations increase during summer and decrease with water depth. All these facts indicate the important role of MAAs as photoprotective agents (Karsten et al. 2006, Ayoub et al. 2012, Tartarotti et al. 2017). Monitoring the MAA pattern is therefore an important task, since it can easily be related to ecological and chemosystematic questions. The preferred technique for MAA analysis is high‐performance liquid chromatography (HPLC), and several methodological approaches have been reported already (Carignan et al. 2009, Rastogi and Incharoensakdi 2014, Hartmann et al. 2015b). This study, however, had two goals: first, to improve the current HPLC methodology to enable the assignment of a larger number of MAAs, and second, to screen different red algae for the occurrence of these, partly new, compounds for the first time.

## Materials and Methods

### Biological material

All of the red algae investigated were collected and morphologically identified by the third author U. Karsten, Prof. J. A. West, University of Melbourne, Australia or Prof. G. C. Zuccarello, Victoria University of Wellington, New Zealand, using their taxonomic expert knowledge in conjunction with standard identification keys (Hiscock 1986; http://www.algaebase.org/); details regarding species, collection date and place are summarized in Table S1 in the Supporting Information. *Palythoa tuberculosa*, a zoanthid, was collected in KB Channel ‐ east side, in Palau, at depths of 17–20 m in February 2018 and identified by Prof. J. D. Reimer, University of the Ryukyus, Japan. Voucher samples of all specimens are deposited at the Institute of Pharmacy, Pharmacognosy, University of Innsbruck, Austria.

### Chemicals and reagents

All solvents which are required for extraction and isolation were purchased from VWR International (Vienna, Austria), and ethyl acetate was distilled before use. Solvents for analytical experiments had pro analysis (p.a.) quality at least and were obtained from Merck (Darmstadt, Germany). Deuterated solvents were supplied by Euriso‐Top (Saint‐Aubin Cedex, France). Ultrapure water was produced by a Sartorius arium^®^ 611 UV (Göttingen, Germany) purification system. Silica gel 40–63 μm and pre‐packed cartridges for flash chromatography were purchased from Merck (Darmstadt, Germany) and Büchi (Flawil, Switzerland), respectively.

### MAA isolation

Three red algae, namely *Pyropia plicata*,* Champia novae‐zelandiae,* and *Agarophyton chilense*, were selected for the isolation of individual MAAs. Either the methanol soluble part of the water extract (*P. plicata*) or the methanolic extract (*A. chilense* and *C. novae‐zelandiae*) was used for further purification. By combining different techniques like silica gel column chromatography, flash chromatography using C‐18 material, semi‐preparative HPLC on diverse stationary phases and fast centrifugal partition chromatography (FCPC), eleven pure MAAs were obtained as standard compounds. The following compounds were isolated from the individual species: the fractionation of *P. plicata* led to the isolation of the compounds 1, 2, 4, 5, 6, 7 and 9, from *C. novae‐zelandiae* compounds 3 and 8 were obtained, while fractionation of the extract of *A. chilense* led to the compounds 5, 6, 10 and 11. Their MS data and nuclear magnetic resonance (NMR) shift values are described as supplementary information (Appendix S1 in the Supporting Information). Original NMR spectra are available upon request, for compound 5 they can be found in the supplementary material.

The methanol soluble part of the aqueous extract of *Pyropia plicata* (54 g) was fractionated on silica gel using gradient elution (EtOAc to methanol), resulting in 18 fractions. Fractions 11, 12, and 14 were individually fractionated with flash chromatography, using a C‐18 40 g cartridge from Büchi and a Reveleris^®^ X2 iES flash chromatography system (Büchi, Flawil, Switzerland). Elution was carried out by the following water (A) and MeOH (B) gradient: 0–7 min: 0% B, 17 min: 20% B, 22 min: 100% B, 22–80 min: 100% B. The flow rate was 15 mL · min^−1^ and UV detection was performed at 254, 310, and 350 nm. Flash chromatography of fraction 12 (1.7 g) directly resulted in the isolation of compound 5 (1 mg) and compound 9 (2 mg). The other two fractions required an additional purification step. Sub‐fraction 11a (300 mg) was subjected to semi‐preparative HPLC using a Triart‐Actus C18 column (150 mm × 20 mm, 5 μm; YMC, Dinslaken, Germany) and a Dionex UltiMate 3000 HPLC (Thermo, Waltham, MA, USA). The mobile phase comprised of 0.25% (v/v) formic acid in water (A) and methanol (B) and the following gradient was used: 0 min: 2% B, 5 min: 10% B, 25 min: 20% B, 30 min: 98% B, 35 min: 98% B. This resulted in the isolation of compounds 2 (2 mg) and 6 (1.5 mg). Sub‐fraction 14a (200 mg) was subjected to semi‐preparative HPLC on a Synergi 4 u Polar‐RP (250 mm × 10 mm, 4 μm; Phenomenex, Torrance, CA, USA) column, using a mobile phase comprising of 0.25% (v/v) formic acid in water (A) and methanol (B); the applied gradient was 0 min: 2% B, 5 min: 10% B, 25 min: 20% B, 30 min: 98% B. This step resulted in the isolation of compounds 1 (150 mg), 4 (100 mg), and 7 (5 mg).

The methanol soluble part of the aqueous extract of *Champia novae‐zelandiae* (16 g) was first fractionated on a silica gel column in gradient mode with EtOAc and methanol as solvents, to give 9 fractions. Fraction 6 (1.7 g) was further purified with flash chromatography, using a C‐18 40 g cartridge (Büchi) and a water/methanol gradient. The so obtained sub‐fraction 6f (20 mg) was purified by semi‐preparative HPLC on a Synergi 4u Polar‐RP (250 mm × 10 mm, 4 μm; Phenomenex) column under isocratic conditions (2% MeOH in 0.25% formic acid) to obtain compound 3 (2 mg). In a next step, sub‐fraction 6d (80 mg) was separated using semi‐preparative HPLC with an Aqua C18 column (250 mm × 10 mm, 5 μm; Phenomenex). The mobile phase was comprised of 0.25% (v/v) formic acid in water (A) and methanol (B), utilized with a gradient of 0 min: 2% B, 5 min: 10% B, 25 min: 20% B. The separation resulted in the isolation of compound 8 (2 mg).

Fast centrifugal partition chromatography was used for the fractionation of the methanol extract of *Agarophyton chilense* (9 g) on an instrument from Kromaton (Annonay, France), equipped with a 200 mL rotor. The system was operated in ascending or descending mode, and a 20 mL injection loop was installed. The experiment was repeated three times due to the maximum amount of 3 g of extract which could be injected.

The applied two phase solvent systems were selected according to the respective partition coefficients (K‐value), which were estimated based on TLC experiments. The following solvent systems were utilized: Solvent System 1: heptane/ethyl acetate/methanol/water = 2/4/1/5 (v/v), Solvent System 2: heptane/ethyl acetate/butanol/methanol/water = 1/4/0.5/1/5 (v/v), Solvent System 3: heptane/ethyl acetate/butanol/methanol/water = 0.5/4/1.5/1/5 (v/v), Solvent System 4: heptane/ethyl acetate/butanol/methanol/water = 0.5/3/2.5/1/5 (v/v), Solvent System 5: heptane/ethyl acetate/butanol/methanol/water = 0.5/2/4.5/1/5 (v/v), and Solvent System 6: heptane/ethyl acetate/butanol/methanol/water = 0.5/1/5.5/1/5 (v/v).

The resulting two phases of each solvent mixture were separated directly before use. The upper organic phase served as the mobile phase, whereas the lower phase was employed as the stationary phase; the consecutive FCPC experiments were conducted in ascending mode, at a flow rate of 5 mL · min^−1^ and 910 rpm rotation speed. The extract was dissolved in a 1:1 (v/v) mixture of the biphasic system at a concentration of 0.16 g · mL^−1^. Then, sequential pumping of the upper mobile phases of each solvent system beginning with SS1 was performed in volumes of 200 mL (elution step); the collected fraction size was 10 mL. The extrusion step was initiated in ascending mode using the lower phase solvent as the mobile phase to ensure that any residual extract was recovered from the apparatus.

All of the obtained fractions were checked by TLC (silica gel plates, chloroform/acetic acid/methanol/water = 15/8/3/2 (v/v) as mobile phase), and those with a similar composition were combined; this resulted in 12 collective fractions. Fraction 5 (20 mg) was subjected to semi‐preparative HPLC using a Triart‐Actus C‐18 column (150 mm × 20 mm, 5 μm; YMC). The mobile phase comprised of 0.25% (v/v) formic acid in water (A) and methanol (B) and the gradient used was 0 min: 2% B, 2 min: 8% B, 47 min: 10% B, and 50 min: 98% B. Compound 10 (2 mg) and compound 11 (2 mg) were isolated in this step. Fraction 10 (3 g) was further fractionated by flash chromatography using a HP 12 g silica cartridge (Büchi) and an ethyl acetate/methanol/ammonia gradient. The so obtained sub‐fraction 10e (200 mg) was subjected to semi‐preparative HPLC using a Synergi 4u Polar‐RP (250 mm × 10 mm, 4 μm; Phenomenex) column in combination with a mobile phase comprising 0.25% (v/v) formic acid in water (A) and methanol (B). By applying gradient elution (0 min: 2% B, 5 min: 12% B, 25 min: 18% B) compounds 5 (2 mg) and 6 (1 mg) were obtained.

### MAA extraction and sample preparation for HPLC analysis

All species that were available in larger quantity (more than 20 g) were crushed to powder in a grinding mill and extracted thrice in an ultrasonic bath (Bandelin Sonorex 35 kHz, Berlin, Germany) for 15 min using dichloromethane. This extract was disposed. For MAA extraction the remaining dry plant material was first extracted using pure methanol under the same conditions, followed by a 3‐fold extraction with methanol/water = 1/1 (v/v). This extract was centrifuged at 1,000 *g* for 6 min and evaporated at 40°C. Subsequently both extracts were combined and freeze‐dried (Heto Powerdry 6000; Thermo). Then the residue was re‐dissolved in methanol (200 mg · mL^−1^) in order to remove a precipitate which contained sugars and salts.

Since a bigger amount of these extracts was available, a more detailed MAA profiling was attempted after performing a single concentration step. Accordingly, 300 mg of the methanol soluble part of each extract were purified on a 12 g HP silica 20 μm Reveleris cartridge using a mobile phase consisting EtOAc (A) and methanol (B). The gradient was as follows: 0 min: 0% B, 6 min: 10% B, 24 min: 100% B and 54 min: 100% B. For each extract four fractions were collected, fraction 1 (0.0–8.0 min), fraction 2 (8.1–17.0 min), fraction 3 (17.1–29.0 min), and fraction 4 (29.1–48.0 min); all fractions were evaporated and used for MAA screening, using a final concentration of 1 mg · mL^−1^ in water.

For species that were only available in small amounts (less than 20 g) a different extraction protocol had to be applied. Respective samples were placed into a shaking flask which was cooled with liquid nitrogen and grinded in a Mikro‐Dismembrator S 8531722 from Sartorius (Göttingen, Germany). The obtained powder was extracted thrice with 1 mL of dichloromethane (this extract was disposed), methanol and methanol/water = 1/1 (v/v) each by sonication. The solutions were centrifuged at 1,000*g* for 6 min, the supernatants combined and dried under an air‐stream due to the small volume. Then, they were dissolved in water to yield a concentration of 1 mg · mL^−1^, membrane filtered (0.45 μm, regenerated cellulose, Phenex, Phenomenex) and directly analyzed by LC/MS.

### Analytical method for MAA separation

The separation of all 11 standard compounds was performed on a YMC‐Pack ODS column (250 mm × 4.60 mm, 5 μm) from YMC, using a mobile phase comprising 20 mM ammonium formate and 0.6% (v/v) formic acid in water (A) and methanol (B). Liquid chromatography ‐ diode array detector ‐ electrospray ionization ‐ mass spectrometry (LC‐DAD‐ESI‐MS) experiments were performed on an Agilent 1260 HPLC system (Santa Clara, CA, USA), which was coupled to an amaZon iontrap mass spectrometer (Bruker, Bremen, Germany). The HPLC instrument was equipped with binary pump, autosampler, column oven and diode array detector.

Elution was performed by maintaining 2% B for the first 15 min, followed by an increase to 10% B in 8 min, to 15% B in 7 min, and to 98% B in further 5 min; this composition was kept for additional 5 min, resulting in a total runtime of 35 min. The column was re‐equilibrated for 15 min prior to the next analysis. The DAD was set to 310, 330, and 350 nm, the flow rate, injection volume, and column temperature were adjusted to 0.65 mL · min^−1^, 5 μL, and 20°C. MS‐spectra were recorded in positive‐ESI mode (capillary voltage 4.5 kV), with a drying gas temperature of 200°C, the nebulizer gas (nitrogen) set to 4.4 psi, and a nebulizer flow (nitrogen) of 6 L · min^−1^. The scanned mass range was set between *m/z* 100 and 1,200.

## Results

### Structural elucidation of MAAs

**Figure 1:**
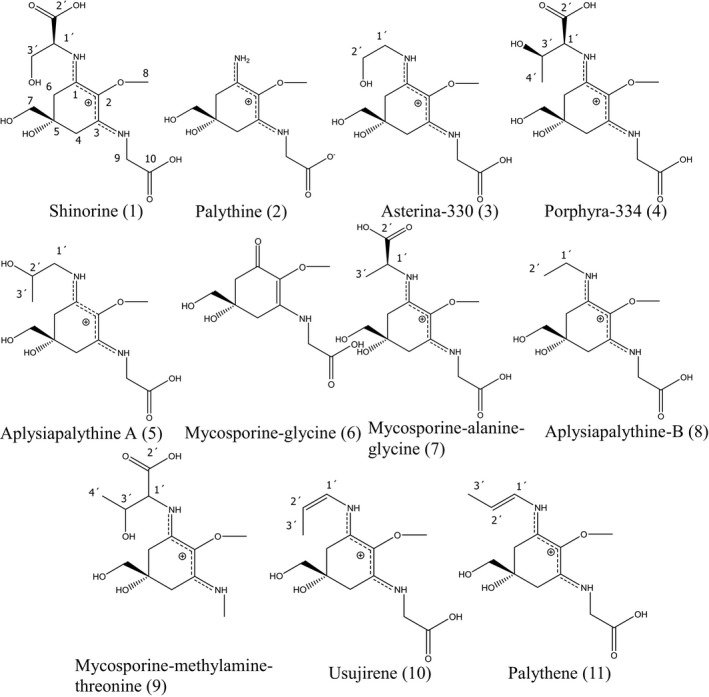
Structures of the isolated MAAs.

Structure elucidation of the isolated compounds was carried out using NMR, UV/vis spectra, and mass spectroscopy, as well as by comparison with already published data. NMR spectra were recorded on a Bruker Ultrashield plus 600 spectrometer operated at 600.19 (^1^H) and 150.91 MHz (^13^C). Chemical shifts are reported in ppm, coupling constants (J) in Hertz. All samples were dissolved in deuterated water (D_2_O) containing tetramethylsilane (TMS) as internal standard. The obtained NMR shifts were in good agreement to the reported values, so that the compounds (shown in Fig. 1) could be identified as follows: shinorine (1; Simon et al. 2014), palythine (2; Takano et al. 1978b), asterina‐330 (3; Nakamura et al. 1981), porphyra‐334 (4; Klisch et al. 2007), aplysiapalythine A (5; Kamio et al. 2011), mycosporine‐glycine (6; Ito and Hirata 1977), mycosporine‐alanine‐glycine (7; Miyamoto et al. 2014), aplysiapalythine B (8; Kamio et al. 2011), mycosporine‐methylamine‐threonine (9; Won et al. 1995), usujirene (10; Nakayama et al. 1999) and palythene (11; Takano et al. 1978a). Appendix S1 compiles all recorded NMR and MS data.

Aplysiapalythine A (5) was difficult to distinguish from palythinol (another known MAA) only by 1D‐NMR and LC‐MS, because both only differ in the location of one methyl group, which is either in position 2′ (aplysiapalythine A) or 1′ (palythinol) of the side chain. Therefore, both compounds show identical molecular masses (*m/z* = 302.10) and absorption maxima, as well as highly similar NMR shift values. By analyzing a sample reported to contain palythinol, the zoanthid *Palythoa tuberculosa* (Takano et al. 1978a), by LC‐MS this similarity became clear. In the corresponding chromatogram, a signal that was identical to the retention time and molecular mass of aplysiapalythine A was visible (Fig. S1 in the Supporting Information). Additionally, in this study the correct structural assignment of the isolated compound 5 was confirmed in 2D‐NMR experiments, where the long‐range correlation from the protons of the methylene group in position 1′ (dd, 3.42 ppm and dd, 3.50 ppm) to carbon 1 of the cyclohexenimine ring confirmed our assumption. The nuclear overhauser enhancement spectroscopy (NOESY) spectrum was helpful as well, since there was a visible correlation from the protons of C‐1′ to the methylene group in position C‐6 (2H, s, 2.87 ppm). For the original NMR spectra of compound 5 see Figures [Link], [Link], [Link], [Link], [Link], [Link], [Link] in the Supporting Information.

### HPLC method development

For the development of an improved HPLC method, all isolated eleven MAAs were used as standards. Four different stationary phases were selected for an initial screening, two from Phenomenex (Synergi MAX‐RP 80A, 150 mm × 4.60 mm, 4 μm; Gemini C18 110A, 150 mm × 4.60 mm, 3 μm) and two from YMC (Triart C18, 150 mm × 3.00 mm, 3 μm; YMC‐Pack ODS column, 250 mm × 4.60 mm, 5 μm). The latter two yielded better results, the Triart C18 phase in respect to a slightly improved peak shape and the YMC‐Pack ODS column indicating overall better separation efficiency; thus it was selected for further experiments.

Acetonitrile and water as mobile phase resulted in poor retention of the analytes and asymmetric peaks, thus methanol was used instead of acetonitrile. The addition of ammonia did not improve the resolution in contrast to acidic modifiers like acetic acid, formic acid or trifluoroacetic acid (TFA), although some of the analytes were still overlapping. Supplementing ammonium formate to the mobile phase showed to be advantageous, and with the finally selected concentration of 20 mM the best peak symmetry and resolution were achieved. Furthermore, a pH value higher than the selected one (pH 2.6 adjusted with formic acid; TFA was not considered because of ion suppression in MS) decreased selectivity and resolution again. In addition, a reduction in the flow rate to 0.65 mL · min^−1^ was required to resolve compounds 8 and 9. For the same reason column temperature was set at 20°C.

**Figure 2:**
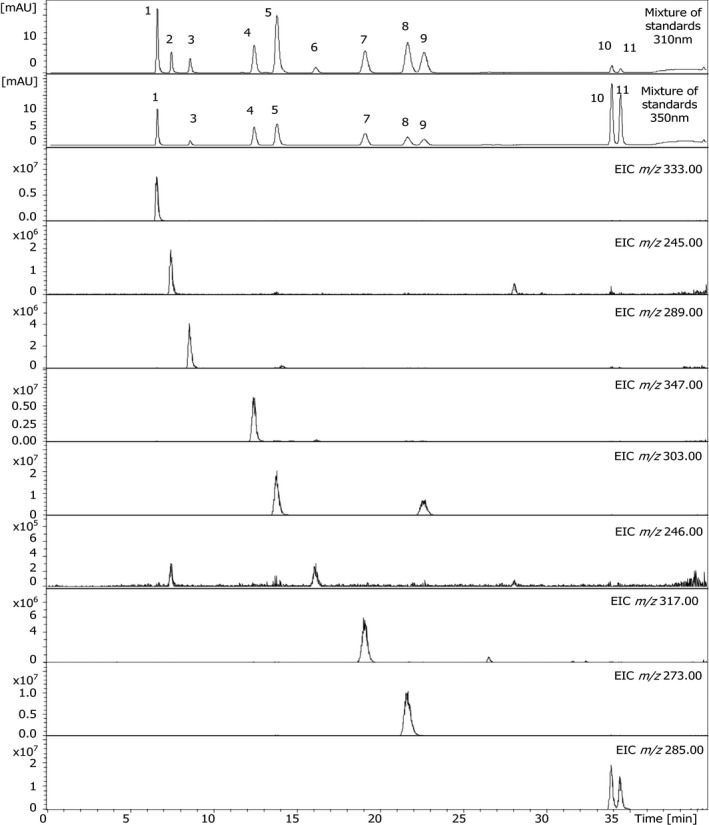
HPLC‐UV‐MS separation of a mixture of eleven mycosporine‐like amino acids under optimized conditions. Peak assignment is according to Figure 1.

Under optimum conditions shinorine (1) eluted first (6.6 min, λ_max_ = 332 nm), followed by palythine (2; 7.5 min, λ_max_ = 320 nm), asterina‐330 (3; 8.6 min, λ_max_ = 330 nm), porphyra‐334 (4; 12.4 min, λ_max_ = 332 nm), aplysiapalythine A (5; 13.8 min, λ_max_ = 330 nm), mycosporine‐glycine (6; 16.1 min, λ_max_ = 310 nm), mycosporine‐alanine‐glycine (7; 19.1 min, λ_max_ = 330 nm), aplysiapalythine B (8; 21.7 min, λ_max_ = 328 nm), mycosporine‐methylamine‐threonine (9; 22.7 min, λ_max_ = 328 nm), usujirene (10; 33.9 min, λ_max_ = 356 nm), and palythene (11; 34.5 min, λ_max_ = 360 nm). In Figure 2 the separation of a mixture of the isolated mycosporine‐like amino acids under optimized conditions is shown together with extracted ion chromatograms (EIC), corresponding to the individual *m/z* values equal to [M+H]^+^. Aplysiapalythine A and mycosporine‐methylamine‐threonine share the same mass (*m/z* = 303), as well as usujirene and palythene (*m/z* = 285); hence two signals are visible. The EIC of mycosporine‐glycine (*m/z* = 246) also shows a second signal, which is due to the isotopic peak of palythine.

### Analysis of mycosporine‐like amino acids in diverse red algae

All of the red algae that were examined contained MAAs. Table 1 lists the respective results divided in two groups, namely species available in small amounts (direct analysis of methanol/water extract) and samples available in larger amounts (analysis of extract after purification step). Individual MAAs could either be assigned by UV/vis (indicated in the table as UV) or only by MS (indicated as T, trace). Shinorine, palythine, and porphyra‐334 were present in most of the samples, and except of a few cases they could be directly assigned by UV; therefore, they definitely are quantitatively dominant UV‐absorbing molecules. Less abundant compounds were asterina‐330 (in 78% of the samples), mycosporine‐glycine (34%), usujirene (26%) and palythene (17%); nevertheless, in most species they still were directly detectable due to matching UV spectra and retention times; however, the correct assignment was also confirmed by LC‐MS.

**Table 1:** Occurrence of MAAs in selected red algae; UV stands for assignment by UV spectra, T stands for trace amounts, identified by LC‐MS after sample cleanup. Assignment of compounds: SH, Shinorine; PA, Palythine; AS, Asterina‐330; PO, Porphyra‐334; APA, Aplysiapalythine A; MG, Mycosporine‐Glycine; MAG, Mycosporine‐Alanine‐Glycine; APB, Aplysiapalythine B; MMT, Mycosporine‐Methylamine‐Threonine; US, Usujirene and PE, Palythene

Species	SH	PA	AS	PO	APA	MG	MAG	APB	MMT	US	PE
(A) Red algae available in large amount (more than 20 g)											
*Pyropia columbina*	UV	UV	T	UV	T^a^		UV	T	T		
*Porphyra umbilicalis*	UV	UV	T	UV	T^a^		UV	T	T		
*Pyropia plicata*	UV	UV	T	UV	UV	UV	UV	T	UV	UV	UV
*Euptilota formosissima*	UV	UV	UV	UV	T^a^						
*Ceramium* sp.	UV	UV	T	UV	T^a^	UV	T		T	T	
*Spongoclonium pastorale*	UV	T	T	UV	T^a^	T	T		T		
*Pterocladia* sp.	UV	UV		UV							
*Agarophyton chilense*	UV	UV	UV	UV	UV	UV	T	T	T	UV	UV
*Schizymenia apoda*	UV	UV		UV		UV					
*Mastocarpus stellatus*	UV	UV	T	T	T^a^		T				
*Sarcothalia atropurpurea*	UV	UV	UV	UV	UV^a^	T	T	T	T	UV	UV
*Gigartina macrocarpa*	UV	UV	UV	UV	T^a^		T	T		T	T
*Rhodophyllis membranecea*	UV	UV	T	UV	T^a^		T			UV	
*Champia novae‐zelandiae*	UV	UV	UV	UV		UV	T	T			
(B) Red algae available in small amount (less than 20 g)											
*Craspedocarpus erosus*	UV	UV	UV	T							
*Blastophyllis calliblepharoides*		T		UV		UV					
*Pachymenia laciniata*	UV	UV	T	T							
*Pterocladia lucida*	UV										
*Corallina officinalis*	UV	UV	UV	UV							
*Gracilariopsis longissima*	UV	T	UV								
*Gracilaria cornea*	UV	T	UV								
*Hymenena affinis*		UV		UV							
*Bostrychia arbuscula*	UV	UV	UV	UV	UV^a^						

a Tentative assignment because palythinol shows identical mass, retention time and UV spectra.

**Figure 3:**
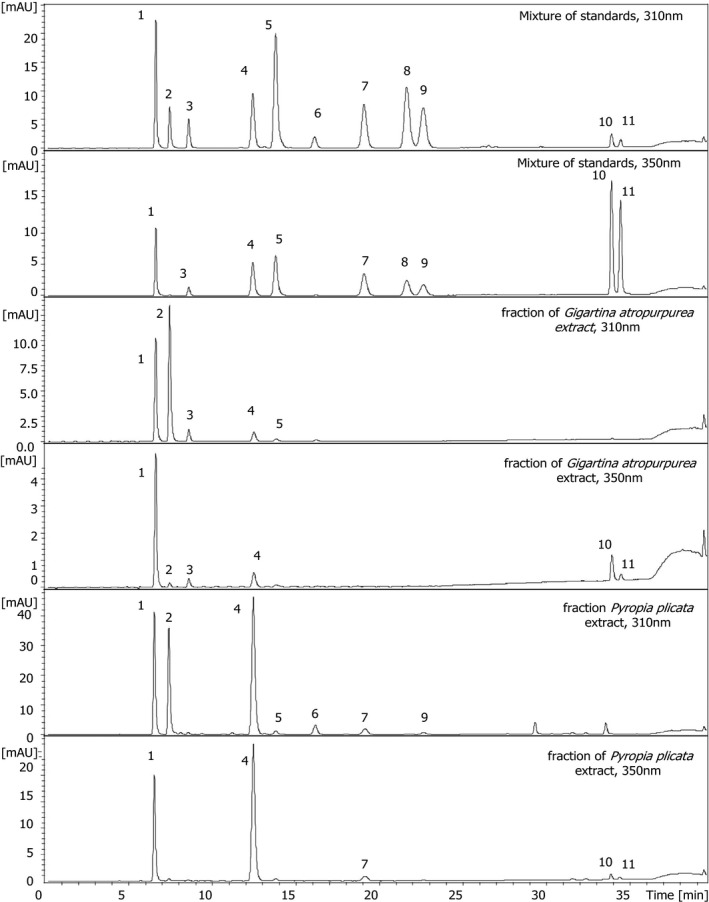
Determination of MAAs in purified fractions of *Sarcothalia atropurpurea* and *Pyropia plicata* in comparison to a standard mixture. Peak assignment according to Figure 1, separation conditions according to Figure 2.

Approximately half of the investigated algae produced aplysiapalythine A and mycosporine‐alanine‐glycine, but together with aplysiapalythine B and mycosporine‐methylamine‐threonine these compounds could mainly be assigned in the MAA‐enriched extracts only. This indicates that they were present in minute concentrations, which is a possible explanation why these four metabolites are reported in our study for the first time in algae. In three of the investigated species (i.e., *Pyropia plicata*,* Agarophyton chilense*, and *Sarcothalia atropurpurea*) all of the standard compounds were present; in *P. plicata* nine out of eleven compounds were assignable in the UV‐chromatogram already (Fig. 3). Samples with the lowest number of identified MAAs were (naturally) those which were analyzed without sample clean‐up. For example, in *Pterocladia lucida* only shinorine could be confirmed, or in *Hymenena affinis* palythine and porphyra‐334 were detected.

## Discussion

Marine organisms are an excellent source for ecologically and pharmacologically relevant natural products. Among them are mycosporine‐like amino acids, known photoprotectants, which are in the focus of the cosmetic industry due to their possible use as sunscreens. Standardized algal extracts containing these compounds are already commercially available and used for their sun protection properties.

Mycosporine‐like amino acids are also interesting ecologically. Thinning of the stratospheric ozone layer, particularly in the Southern Hemisphere and as reflected in the Antarctic Ozone Hole which resulted in an increase of biologically harmful solar UVR reaching the Earth's surface (Bai et al. 2016) magnifies their importance. The multiple effects of UVR on Rhodophyta and other algae have been studied for decades, and different protective mechanisms against excessive solar radiation were reported (Karsten 2007 and references therein). These include avoidance (e.g., living in the shade of canopy algae and/or in great water depth), numerous physiological and biochemical protective mechanisms (e.g., dynamic photoinhibition, antioxidants, UV‐sunscreens) and repair or de‐novo synthesis of essential biomolecules (e.g., DNA repair; Karsten 2007). The biosynthesis and accumulation of MAAs is a highly efficient photoprotective mechanism. These compounds act as passive shielding solutes by dissipating the absorbed short wavelength radiation energy in the harmless form of heat without generating photochemical reactions (Bandaranayake 1998). MAAs exhibit extremely high‐molar absorptivity for UVA and UVB (molar extinction coefficients between 28,000 and 50,000), and have been shown to be photochemically stable structures, both of which are prerequisites for their sunscreen function (Gleason 1993 and references therein, Conde et al. 2000).

The most frequently used protocols for MAA extraction include the aqueous extraction in an ultrasonic bath followed by filtration to remove debris (Whitehead and Hedges 2002, Carreto et al. 2005), extraction with pure methanol after soaking of the samples with water in the dark at 4°C overnight (Carreto et al. 2005), or extraction of lyophilized samples in 25% aqueous methanol at 45°C for 2 h (Tartarotti and Sommaruga 2002). In our study, a combination of the previously reported methods for extraction was used, which included two further steps: first, extraction with dichloromethane prior to extraction with methanol in order to remove lipophilic components, and second, the combined methanol and methanol/water extracts were re‐dissolved in methanol. This MAA enrichment step resulted in the precipitation and hence removal of sugars and salts, which otherwise would negatively affect the then following HPLC analysis.

Several chromatographic techniques for the isolation of MAAs have been used in the past including gel permeation, ion exchange resins, preparative TLC and HPLC, which all are well summarized in a review by Carreto and Carignan (2011). The main advantage of the protocol used in this study was the lower solvent consumption and reduced duration of the isolation procedure especially when FCPC and flash chromatography were combined (both experiments were completed in a few hours). Furthermore, FCPC is ideal for easy scale up and it requires no solid packing material, which can be quite expensive; furthermore irreversible adsorption or sample loss is avoided (Berthod 2007, Chollet et al. 2015). On the other hand, the main drawback of FCPC is usually a time‐consuming optimization of the biphasic solvent system and the operating conditions.

The isolated MAAs were used as purified standards to develop an improved HPLC method, allowing their qualitative and quantitative determination in a large number of red algal species. All of the isolated compounds were known already from marine organisms; yet, four of them (mycosporine‐methylamine‐threonine, mycosporine‐alanine‐glycine, aplysiapalythine A and aplysiapalythine B) were isolated for the first time from an algal species. The compounds aplysiapalythine A and B were previously reported as constituents of the sea hare *Aplysia californica* (Kamio et al. 2011), and the authors suggested that these animals acquire MAAs from their algal diet. The latter were proven to produce some other mycosporine‐like amino acids but not aplysiapalythine A and aplysiapalythine B in a detectable amount. Mycosporine‐methylamine‐threonine has been isolated from the reef‐building corals *Pocillopora damicornis* and *Stylophora pistillata* (Won et al. 1995), while mycosporine‐alanine‐glycine has only artificially been produced by Actinomycetales through heterologous expression (Miyamoto et al. 2014). Since many marine animals contain MAAs, but lack the biosynthetic capability to produce these compounds, a dietary origin from grazing on algae is the only plausible explanation. Indeed, algal diets can regulate MAA concentration and composition in marine invertebrates and fish (Karentz 2001, Shick and Dunlap 2002). The ingested MAAs are often specifically bioaccumulated in the most UV‐susceptible tissues or reproductive structures (e.g., eggs; Adams and Shick 1996), and can be interconverted to animal‐specific MAAs in the digestive track by animal enzymes or by endosymbiotic bacteria.

The novel HPLC method allows an excellent separation of all 11 standard substances within 35 min and it has several advantages compared to previously published methods. First, none of them permitted the separation of that many MAAs based on pure substances except that of Carreto et al. (2005). Second, despite using a conventional reversed phase column the compounds were adequately retained and their baseline separation was possible. Additionally, due to the use of a volatile mobile phase an MS detector could be used, which allowed the assignment of minor components as well.

For those samples which were analyzed after an enrichment step, a more detailed chemical profiling of the MAA composition was possible. For example, species belonging to the families Bangiaceae, Gigartinaceae, Gracilariaceae, and Ceramiaceae contained almost all 11 MAAs identified in this study, while species from other families such as Schizymeniaceae, Pterocladiaceae, and Callithamniaceae exhibited only less than five compounds. Both species from the genus *Porphyra* had exactly the same MAA pattern, while both species belonging to the genus *Gigartina* differed in two compounds. In conclusion, it is obvious that conspicuous differences in MAA patterns can be observed between the different families. However, whether such detailed MAA patterns as shown in this study can be used for chemotaxonomic purposes in the red algae has to be addressed in follow‐up investigations. At least for green algae within the *Prasiola*‐clade (Trebouxiophyceae), the presence of the MAA prasiolin represents a suitable chemotaxonomic marker (Hotter et al. 2018). Likewise, the possible confusion between aplysiapalythine A and palythinol is due to the fact that both molecules have the same molecular mass and UV spectra (and possibly identical retention times); therefore, their differentiation is not possible using HPLC‐MS. However, NMR data unambiguously confirmed that compound 5 which was isolated in this study from two species (*Pyropia plicata* and *Agarophyton chilense*) is aplysiapalythine A. None of the previous reports contained conclusive NMR data (including two‐dimensional spectra) and therefore the correct assignment of aplysiapalythine A and/or palythinol in the past is questionable. This may also include the first report on the isolation of palythinol from *Palythoa tuberculosa*, an organism which was used for comparison in our study. Accordingly, all published data within this context have to be critically evaluated and future studies need to be aware of this possible pitfall.

In conclusion, our data on MAAs clearly indicate that more of these UV‐sunscreens exist in Rhodophyta and probably other algal groups than previously considered. In addition to their pronounced UV‐protective effect, some MAAs such as mycosporine‐glycine also have moderate antioxidant activity (Dunlap and Yamamoto 1995). The presumed biochemical precursor of MAAs, 4‐deoxygadusol exhibits strong antioxidant activity (Dunlap et al. 2002). Therefore, the photo‐physicochemical properties of MAAs guarantee both a high UV‐protective effectiveness in combination with antioxidant capabilities. Rhodophyta represent excellent model systems to study and understand the underlying mechanisms. With new developments in genomics, proteomics, metabolomics, and analytical chemistry new types of MAAs will continue to be discovered and their biosynthetic and regulatory mechanisms elucidated.

This work was financially supported by the Austrian Science Fund (FWF), project no. P29671. We thank Prof. James Davis Reimer, University of the Ryukyus, for providing the *P. tuberculosa* sample from the Palau International Coral Reef Center (PICRC) which was supported by the SATREPS P‐CoRIE Project “Sustainable management of coral reef and island ecosystem: responding to the threat of climate change,” funded by the Japan Science and Technology Agency (JST) and the Japan International Cooperation Agency (JICA) in cooperation with PICRC and Palau Community College. We would also like to thank Prof. Giuseppe C. Zuccarello, School of Biological Sciences, Victoria University of Wellington, New Zealand and Prof. John A. West, School of Botany, University of Melbourne, Australia, who contributed to the collection and identification of the algal species from New Zealand and other regions.

## Author Contributions

M.O. did all practical work (isolation and elucidation of MAAs, method development, and analysis of samples) and prepared the first draft of the manuscript; U.K. supplied and identified the algae. A.H. and M.G. supervised M.O., helped with all methodological approaches, and finalized the paper along with the other co‐authors.

## Conflicts of Interest

The authors declare no conflict of interest.

## Supporting information


**Figure S1.** Analysis of the methanolic extract of *Palythoa tuberculosa*, an anemone reported to contain the MAA palythinol, by HPLC and LC‐MS in comparison to a standard mixture of eleven MAAs.

**Figure S2.** ^1^H‐NMR spectrum of compound 5, aplysiapalythine A.

**Figure S3.** ^13^C‐NMR spectrum of compound 5, aplysiapalythine A.

**Figure S4.** COSY spectrum of compound 5, aplysiapalythine A.

**Figure S5.** HSQC spectrum of compound 5, aplysiapalythine A.

**Figure S6.** HMBC‐1 spectrum of compound 5, aplysiapalythine A.

**Figure S7.** HMBC‐2 spectrum of compound 5, aplysiapalythine A.

**Figure S8.** NOESY spectrum of compound 5, aplysiapalythine A.

**Table S1.** Overview on the investigated species, their collection sites and dates.

**Appendix S1.** Assignments for the 11 mycosporine‐like amino acids.
