# Supplementary figures and images for: Chemical profiling of mycosporine‐like amino acids in twenty‐three red algal species
Source: J Phycol. 2019 Jan 31;55(2):393–403. doi: 10.1111/jpy.12827 (PMC6492128; doi:10.1111/jpy.12827)

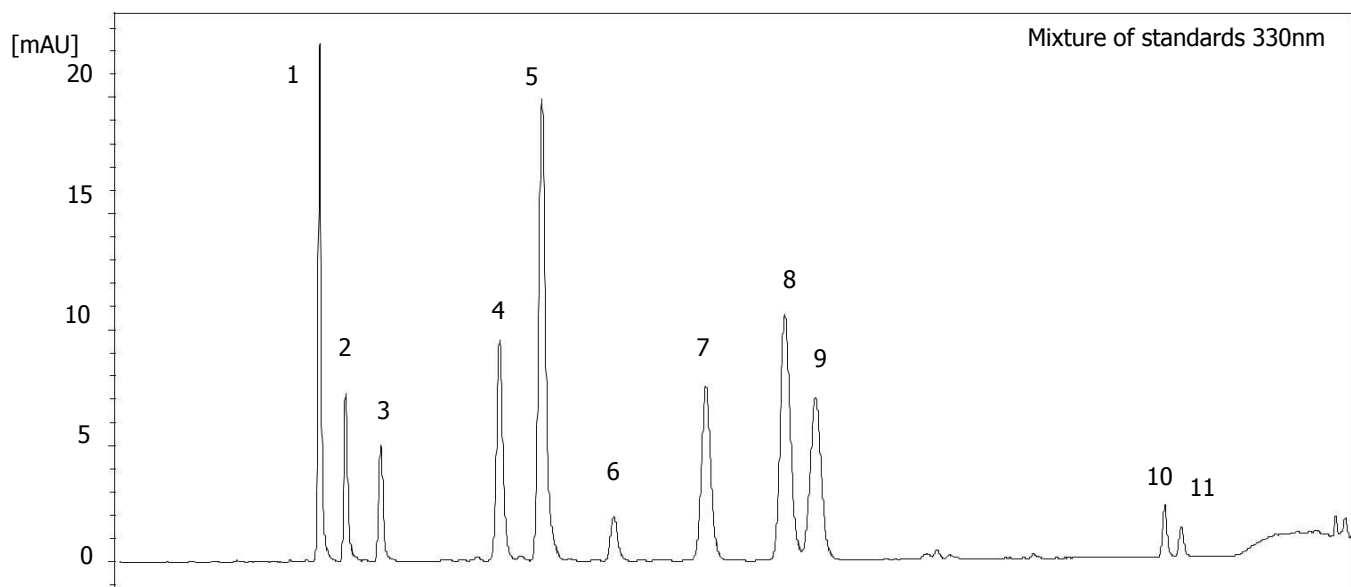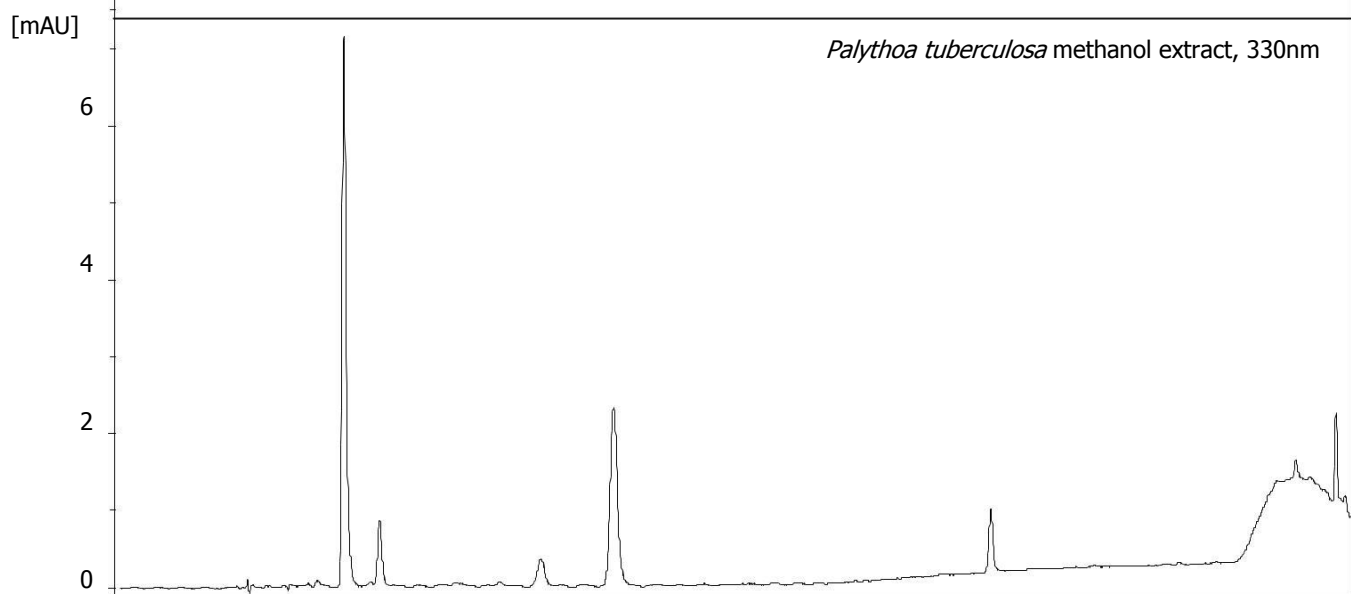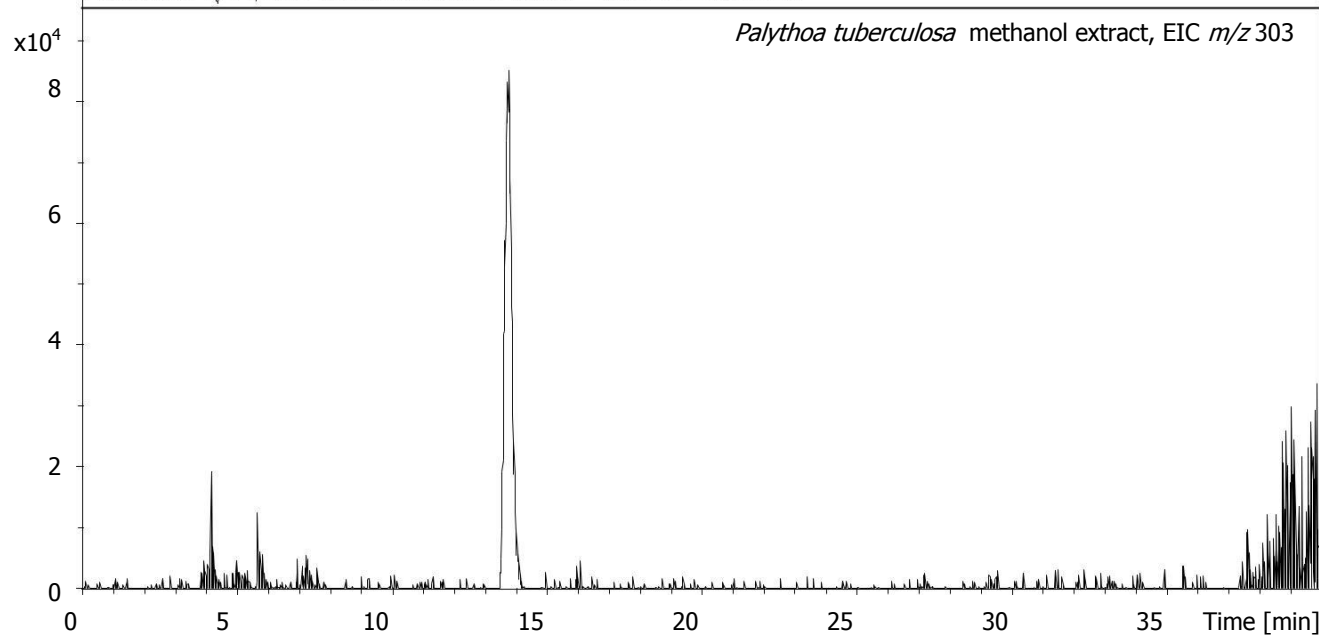

Supplement: Supplementary file 1 — Figure S1. Analysis of the methanolic extract of Palythoa tuberculosa, an anemone reported to contain the MAA palythinol, by HPLC and LC‐MS in comparison to a standard mixture of eleven MAAs. [file JPY-55-393-s001.pdf]

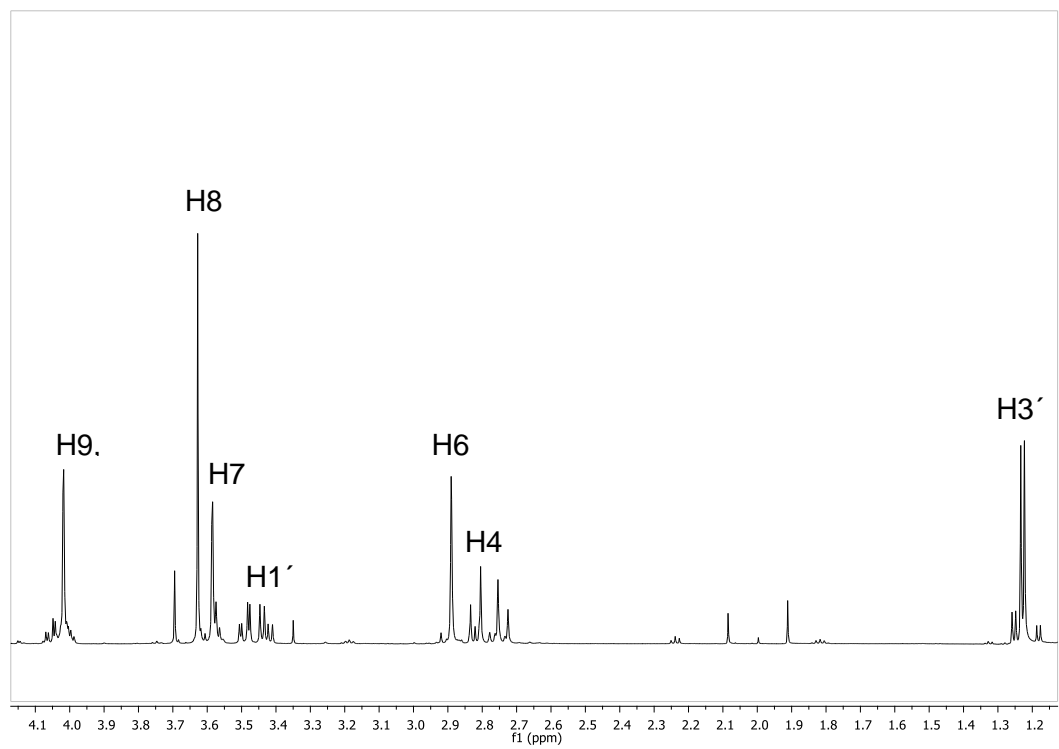

Supplement: Supplementary file 2 — Figure S2. 1H‐NMR spectrum of compound 5, aplysiapalythine A. [file JPY-55-393-s002.pdf]

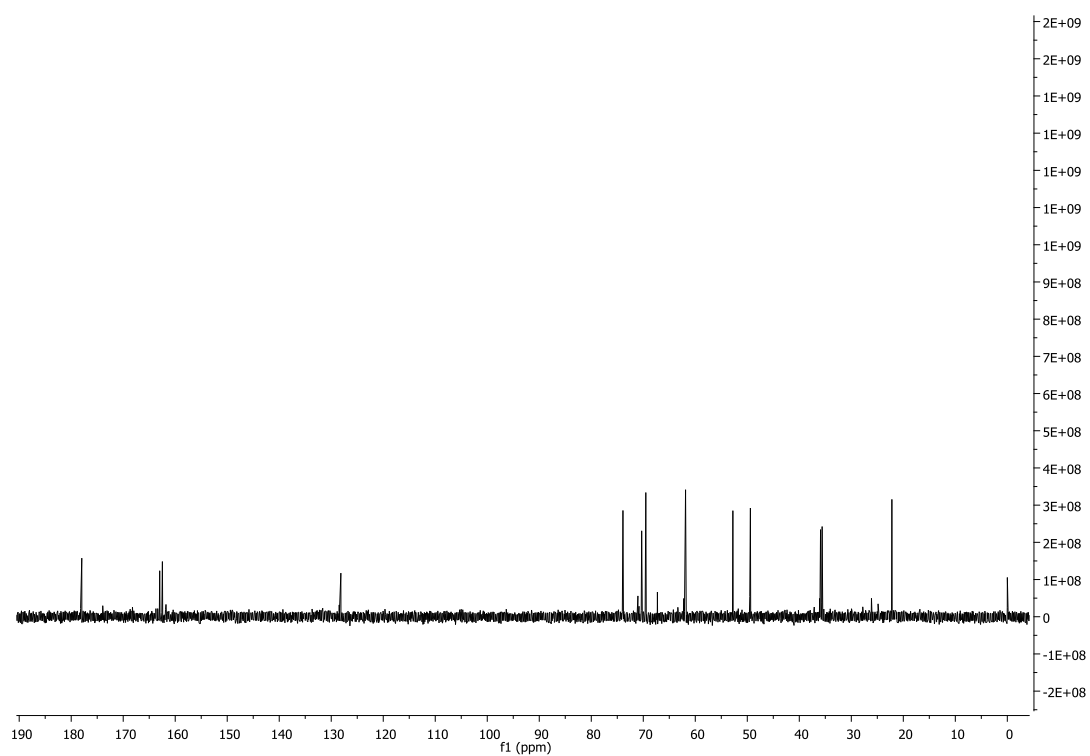

Supplement: Supplementary file 3 — Figure S3. 13C‐NMR spectrum of compound 5, aplysiapalythine A. [file JPY-55-393-s003.pdf]

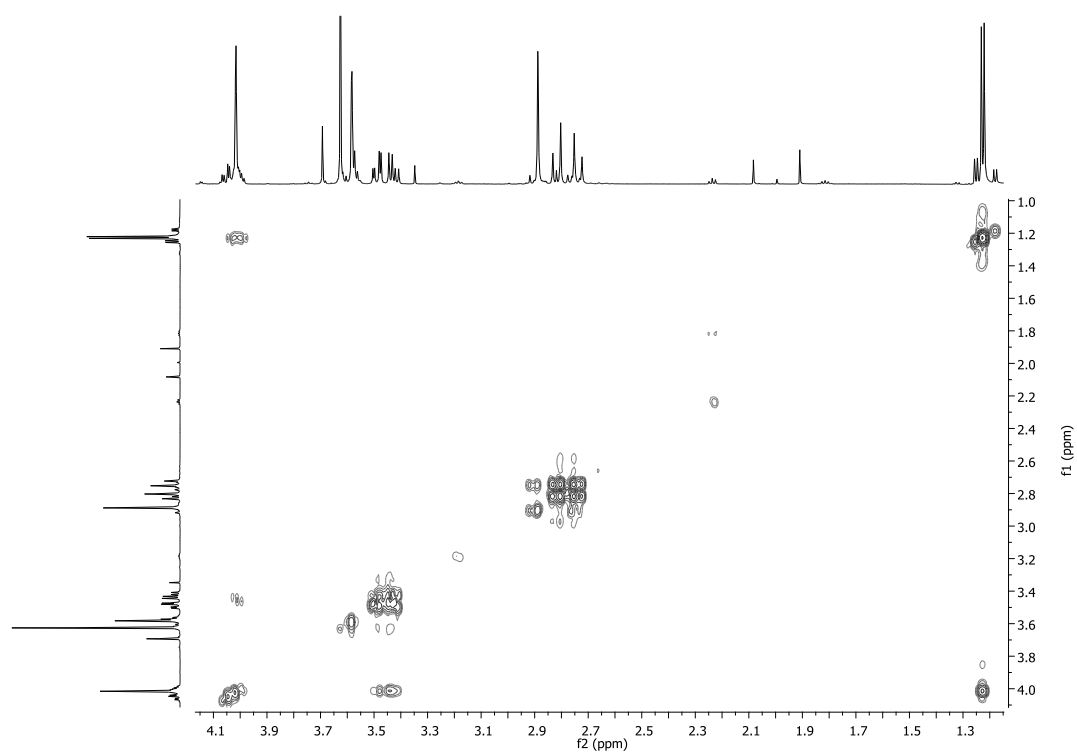

Supplement: Supplementary file 4 — Figure S4. COSY spectrum of compound 5, aplysiapalythine A. [file JPY-55-393-s004.pdf]

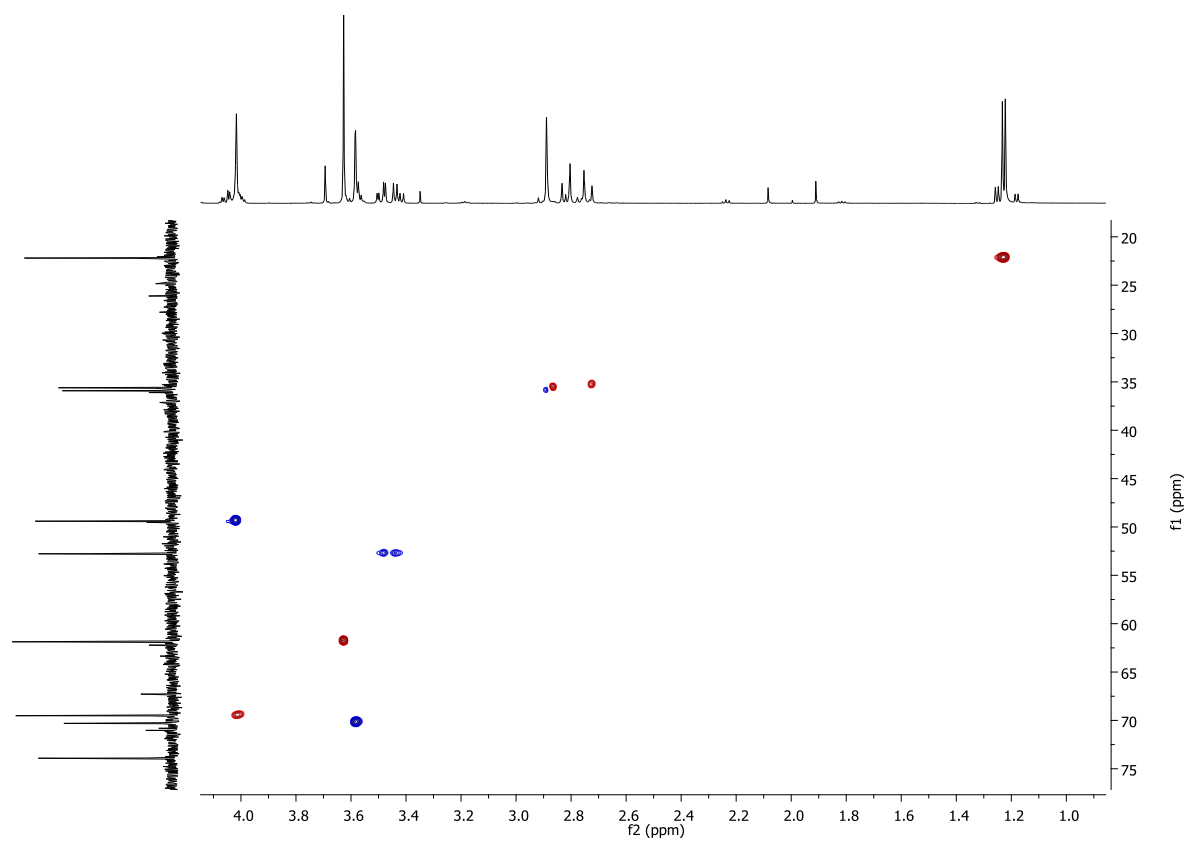

Supplement: Supplementary file 5 — Figure S5. HSQC spectrum of compound 5, aplysiapalythine A. [file JPY-55-393-s005.pdf]

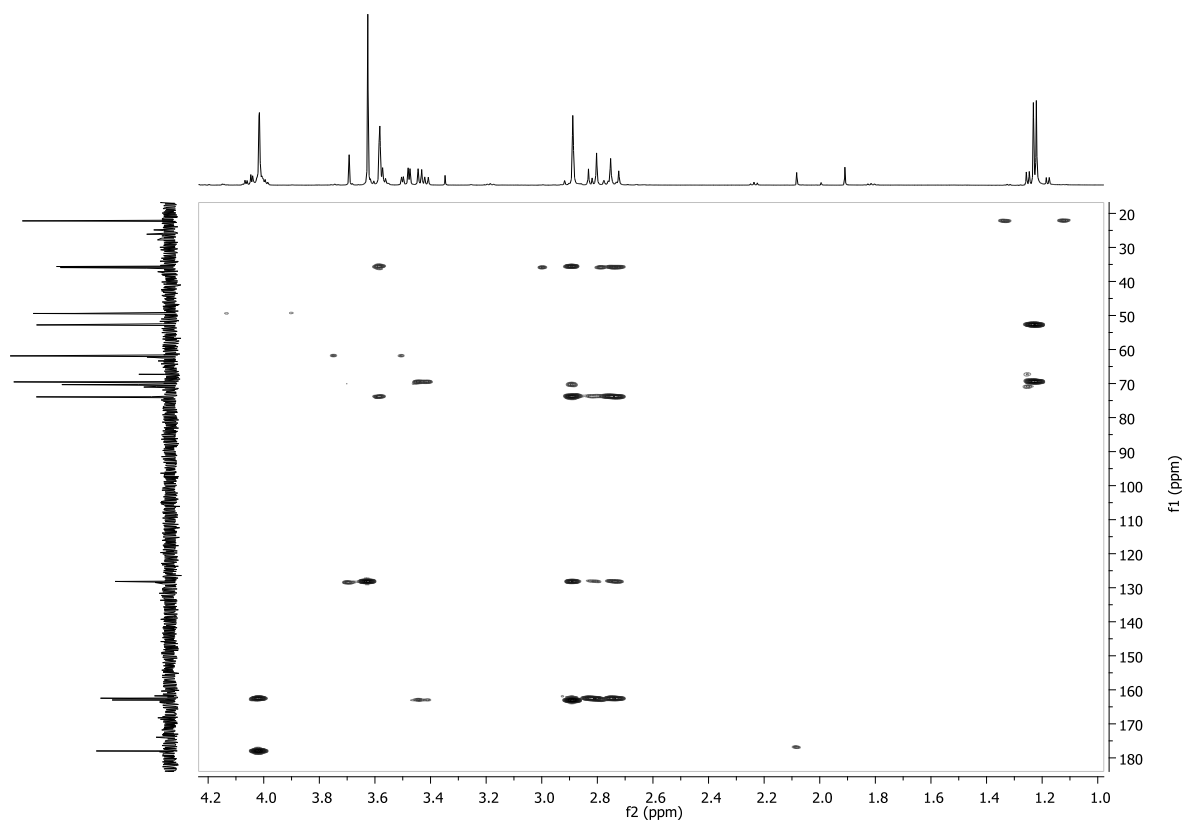

Supplement: Supplementary file 6 — Figure S6. HMBC‐1 spectrum of compound 5, aplysiapalythine A. [file JPY-55-393-s006.pdf]

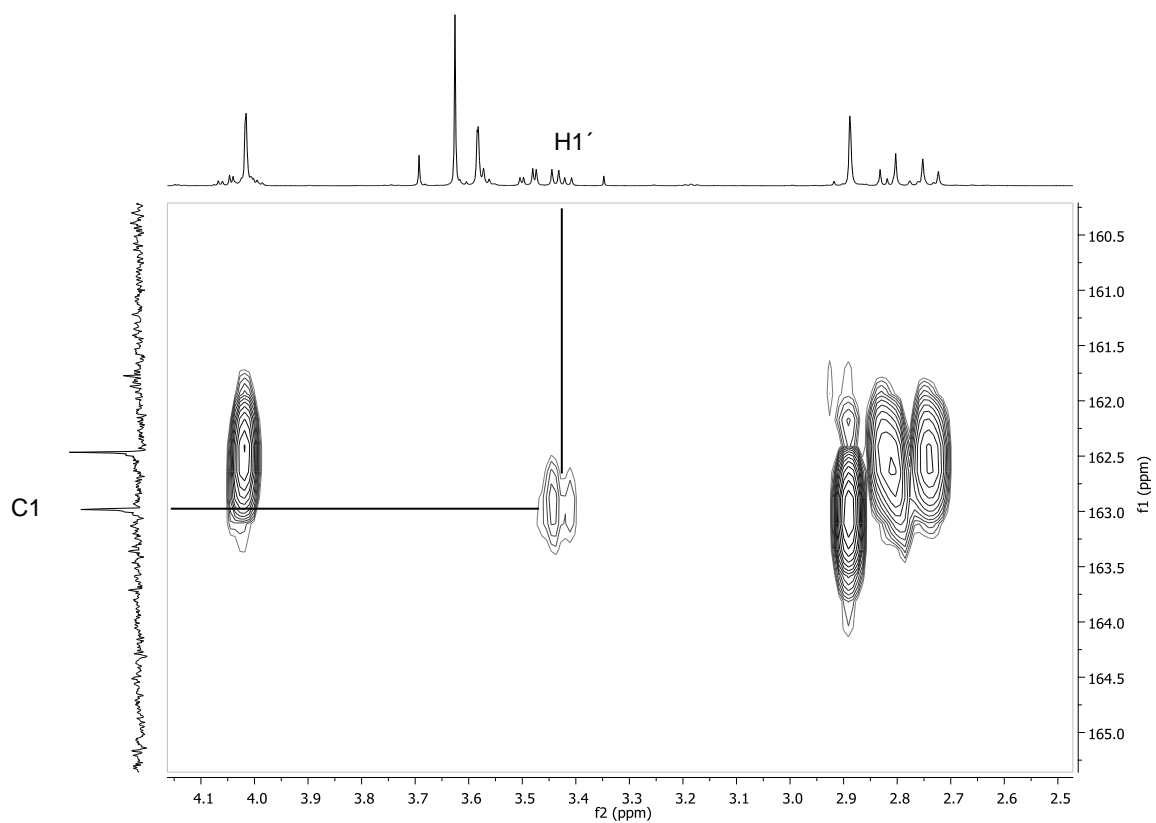

Supplement: Supplementary file 7 — Figure S7. HMBC‐2 spectrum of compound 5, aplysiapalythine A. [file JPY-55-393-s007.pdf]

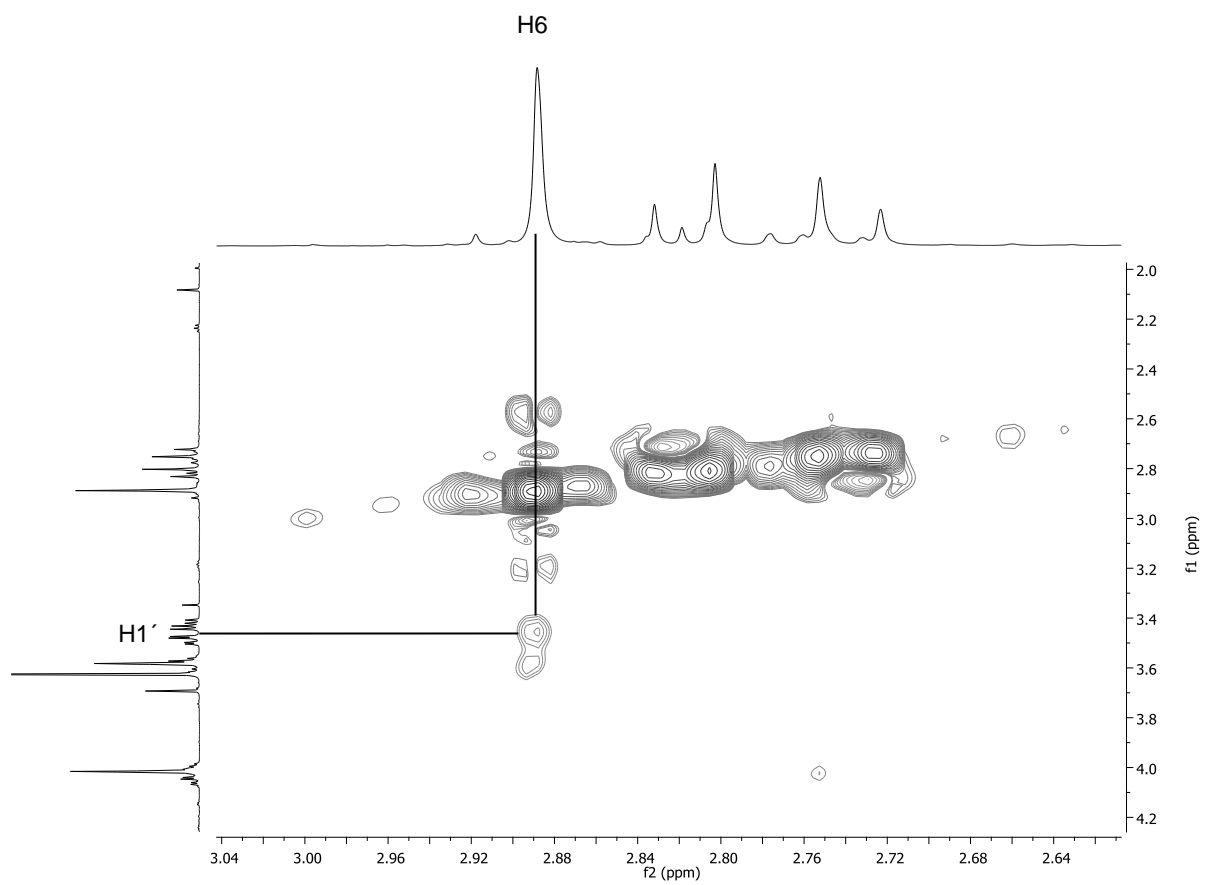

Supplement: Supplementary file 8 — Figure S8. NOESY spectrum of compound 5, aplysiapalythine A. [file JPY-55-393-s008.pdf]
