# Supplementary material for: Chemical profiling of mycosporine‐like amino acids in twenty‐three red algal species
Source: J Phycol. 2019 Jan 31;55(2):393–403. doi: 10.1111/jpy.12827 (PMC6492128; doi:10.1111/jpy.12827)
Supplement: Supplementary file 9 — Table S1. Overview on the investigated species, their collection sites and dates. [file JPY-55-393-s009.pdf]

A) Red algae available in large amount (more than 20g)			
**Species**	**Family**	**Order**	**Collection Place, Date**
Pyropia columbina	Bangiaceae	Bangiales	Sydney, Towra Point, Australia, 1995
Porphyra umbilicalis	Bangiaceae	Bangiales	Helgoland, Harbour, Germany, 1997
Pyropia plicata	Bangiaceae	Bangiales	Wellington Moa Point, New Zealand, 6/11/2016
Euptilota formosissima	Callithamniaceae	Ceramiales	Wellington Moa Point, New Zealand,1/11/2016; 6/11/2016
Ceramium sp.	Ceramiaceae	Ceramiales	Wellington Moa Point, New Zealand,1/11/2016
Spongoclonium pastorale	Ceramiaceae	Ceramiales	Wellington Moa Point, New Zealand,1/11/2016; 6/11/2016
Pterocladia sp.	Pterocladiaceae	Gelidiales	Wellington Princess Bay, New Zealand, July 2016
Agarophyton chilense	Gracilariaceae	Gracilariales	Wellington Scorching Bay, New Zealand, May 2016

Schizymenia apoda	Schizymeniaceae	Nestomatales	Wellington Harbour, New Zealand, 18/11/2016
Mastocarpus stellatus	Phyllophoraceae	Gigartinales	Helgoland, Germany, 2006
Sarcothalia atropurpurea	Gigartinaceae	Gigartinales	Wellington Moa Point, New Zealand, August 2016
Gigartina macrocarpa	Gigartinaceae	Gigartinales	Wellington Evans Bay, New Zealand, August 2016
Rhodophyllis membranecea	Cystocloniaceae	Gigartinales	Wellington Harbour, New Zealand, 1/11/2016; 6/11/2016
Champia novae-zelandiae	Champiaceae	Rhodymeniales	Wellington Moa Point, New Zealand, 6/11/2016

B) Red algae available in small amount (less than 20g)

**Species**	**Family**	**Order**	**Collection Place, Date**
Craspedocarpus erosus	Cystocloniaceae	Gigartinales	Wellington Noa Point, New Zealand, 6/11/2016
Blastophyllis calliblepharoides	Kallymeniaceae	Gigartinales	Wellington Noa Point, New Zealand, 1/11/2016; 6/11/2016
Pachymenia laciniata	Halymeniaceae	Halymeniales	Wellington Harbour, New Zealand, May 2016
Pterocladia lucida	Pterocladiaceae	Gelidiales	Wellington Harbour, New Zealand, 29/09/2016

Corallina officinalis	Corallinaceae	Corallinales	Wellington Harbour, New Zealand, 29/09/2016
Gracilariopsis longissima	Gracilariaceae	Gracilariales	Rio San Pedro, Cádiz, Spain, 2017
Gracilaria cornea	Gracilariaceae	Gracilariales	Gran Canaria (Spanish Bank of algae of Las Plamas GC University), Spain, 2017
Hymenena affinis	Delesseriaceae	Ceramiales	Wellington Noa Point, New Zealand, 1/11/2016; 6/11/2016
Bostrychia arbuscula	Rhodomelaceae	Ceramiales	Dunedin Harbour, New Zealand, November 2004
