# Supplementary material for: Chemical profiling of mycosporine‐like amino acids in twenty‐three red algal species
Source: J Phycol. 2019 Jan 31;55(2):393–403. doi: 10.1111/jpy.12827 (PMC6492128; doi:10.1111/jpy.12827)
Supplement: Supplementary file 10 — Appendix S1. Assignments for the 11 mycosporine‐like amino acids. [file JPY-55-393-s010.pdf]

Assignments for **1** (shinorine): ESI-MS: m/z 333.11 $[M+H]^+$; ^{1}H-NMR (D_2O): δ_H 4.48 (1H, dd, J=4.8, 6.4, H-1´); 4.18 (2H, d, J=3.1, H-9); 4.02 (1H, dd, J=3.6, 12.0, H-3´); 3.96 (1H, dd, J=6.4 ,12.0, H-3´) 3.67 (3H, s, H-8); 3.57 (2H, s, H-7); 2.93 (1H, d, J=17.8, H-6); 2.84 (1H, d, J=17.8, H-4); 2.79 (1H, d, J=17.8, H-4) 2.79 (1H, d, J=17.8, H-6); ^{13}C-NMR (D_2O): δ_C 174.03 (C-2´); 173.43 (C-10); 160.46 (C-3); 159.69 (C-1); 125.58 (C-2); 73.92 (C-5); 71.13 (C-3´); 70.20 (C-7); 67.25 (C-1´); 62.22 (C-8); 49.50 (C-9); 36.13 (C-6); 35.67 (C-4); 22.22 (C-4´).

Assignments for **2** (palythine): ESI-MS: m/z 244.98 $[M+H]^+$; ^{1}H-NMR (D_2O): δ_H 4.03 (2H, s, H-9); 3.64 (3H, s, H-8); 3.57 (2H, s, H-7); 2.95 (1H, d, J=17.8, H-6); 2.83 (1H, d, J=17.8, H-4); 2.75 (1H, d, J=17.8, H-4) 2.68 (1H, d, J=17.8, H-6).

Assignments for **3** (asterina-330): ESI-MS: m/z 289.07 $[M+H]^+$; ^{1}H-NMR (D_2O): δ_H 4.02 (2H, s, H-9); 3.77 (2H, d, J=5.3 Hz, H-1´); 3.65 (3H, s, H-8); δH 3.60 (2H, d, J=5.3 Hz, H-2´); 3.59 (2H, s, H-7); 2.91 (2H, s, H-6); 2.82 (1H, d, J=17.8 Hz, H-4); 2.74 (1H, d, J=17.8 Hz, H-4).

Assignments for **4** (porphyra-334): ESI-MS: m/z 347.12 $[M+H]^+$; ^{1}H-NMR (D_2O): δ_H 4.30 (1H, dq, J= 4.6, 6.4 Hz, H-3´); 4.07 (1H, d, J=4.4 Hz, H-1´); 4.05 (2H, d, J=3.8 Hz, H-9); 3.70 (3H, s, H-8); 3.58 (2H, s, H-7); 2.94 (1H, d, J=17.8 Hz, H-6); 2.83 (1H, d, J=17.8 Hz, H-4); 2.77 (1H, d, J=17.8 Hz, H-4); 2.75 (1H, d, J=17.8 Hz, H-6); 1.26 (3H, d, J=6.4 Hz, H-4´); ^{13}C-NMR (D_2O): δ_C 178.18 (C-2´); 177.73 (C-10); 163.41 (C-3); 162.03 (C-1); 128.53 (C-2); 73.92 (C-5); 71.13 (C-3´); 70.20 (C-7); 67.25 (C-1´); 62.22 (C-8); 49.50 (C-9); 36.13 (C-6); 35.67 (C-4); 22.22 (C-4´).

Assignments for **5** (aplysiapalythine A): ESI-MS: m/z 303.10 $[M+H]^+$; ^{1}H-NMR (D_2O): δ_H 4.02 (2H, d, J=0.9 Hz, H-9); 4.01 (1H, m, H-2´); 3.63 (3H, s, H-8); 3.59 (2H, s, H-7); 3.49 (1H, dd, J=14.0 Hz, 4.0, H-1´); 3.43 (1H, dd, J=14.0 Hz, 7.3, H-1´); 2.89 (2H, s, H-6); 2.82

(1H, d, J=17.8 Hz, H-4); 2.74 (1H, d, J=17.8 Hz, H-4); 1.23 (3H, d, J=6.4 Hz, H-3´); ^{13}C-NMR (D_2O): δ_C 177.84 (C-10); 162.01 (C-3); 162.83 (C-1); 127.85 (C-2); 73.62 (C-5); 70.35 (C-7); 69.55 (C-2´); 61.67 (C-8); 52.72 (C-1´); 49.42 (C-9); 35.61 (C-6); 35.14 (C-4); 21.91 (C-3´).

Assignments for **6** (mycosporine-glycine): ESI-MS: m/z 245.95 $[M+H]^+$; ^{1}H-NMR (D_2O): δ_H 3.98 (2H, s, H-9); 3.59 (3H, s, H-8); 3.53 (2H, s, H-7); 2.83 (1H, d, J=17.8 Hz, H-6); 2.68 (1H, d, J=17.8 Hz, H-6); 2.67 (1H, d, J=17.8 Hz, H-4) 2.43 (1H, d, J=17.8 Hz, H-4); ^{13}C-NMR (D_2O): δ_C 188.24 (C-1); 178.85 (C-10); 161.93 (C-3); 132.44 (C-2); 74.82 (C-5); 70.15 (C-7); 62.39 (C-8); 44.12 (C-6); 43.95 (C-9); 35.44 (C-4).

Assignments for **7** (mycosporine-alanine-glycine): ESI-MS: m/z 317.11 $[M+H]^+$; ^{1}H-NMR (D_2O): δ_H 4.24 (1H, q, J=7 Hz, H-1´); 4.02 (2H, d, J=3.6 Hz, H-9); 3.65 (3H, s, H-8); 3.57 (2H, s, H-7); 2.94 (1H, d, J=17.8 Hz, H-6); 2.80 (1H, d, J=17.8 Hz, H-4); 2.74 (1H, d, J=17.8 Hz, H-4); 2.72 (1H, d, J=17.8 Hz, H-6); 1.26 (3H, d, J=7 Hz, H-3´); ^{13}C-NMR (D_2O): δ_C 181.66 (C-2´); 178.35 (C-10); 163.41 (C-3); 163.16 (C-1); 128.03 (C-2); 73.36 (C-5); 70.06 (C-7); 57.48 (C-1´); 61.82 (C-8); 49.41 (C-9); 36.28 (C-6); 35.95 (C-4); 21.12 (C-3´).

Assignments for **8** (aplysiapalythine B): ESI-MS: m/z 273.07 $[M+H]^+$; ^{1}H-NMR (D_2O): δ_H 4.03 (2H, s, H-9); 3.46 (1H, q, J=7 Hz, H-1´); 3.64 (3H, s, H-8); 3.62 (2H, s, H-7); 2.75-2.94 (4H, d, J=17.8 Hz, H-4 and H-6); 1.27 (3H, t, J=7 Hz, H-2´).

Assignments for **9** (mycosporine-methylamine threonine): ESI-MS: m/z 303.10 $[M+H]^+$; ^{1}H-NMR (D_2O): δ_H 4.29 (1H, dq, J= 4.3, 6.3 Hz, H-3´); 4.03 (1H, d, J=4.3 Hz, H-1´); 3.65 (3H, s, H-8); 3.59 (2H, s, H-7); 3.09 (3H, s, H-9); 2.88 (2H, s, H-6); 2.87 (1H, d, J=17.8 Hz, H-4); 2.72 (1H, d, J=17.8 Hz, H-4); 1.24 (3H, d, J=6.3 Hz, H-4´); ^{13}C-NMR (D_2O): δ_C 175.46 (C-2´); 161.60 (C-3); 158.67 (C-1); 125.07 (C-2); 71.40 (C-5); 68.15 (C-3´); 67.64 (C-7); 64.50 (C-1´); 58.58 (C-8); 33.48 (C-6); 33.48 (C-4); 30.65 (C-9); 19.77 (C-4´).

Assignments for **10** (usujirene): ESI-MS: m/z 285.07 $[M+H]^+$; ^{1}H-NMR (D_2O): δ_H 6.41 (1H, dd, J= 1.3, 7.7 Hz, H-1´); 5.43 (1H, dq, J=7.4, 7.7 Hz, H-2´); 4.11 (2H, s, H-9); 3.72 (3H, s, H-8); 3.63 (2H, s, H-7); 2.83-2.96 (4H, s, H-4 and H-6); 1.79 (3H, dd, J=1.3, 7.4 Hz, H-3´).

Assignments for **11** (palythene): ESI-MS: m/z 285.07 $[M+H]^+$; ^{1}H-NMR (D_2O): δ_H 6.59 (1H, dd, J= 1.3, 13.7 Hz, H-1´); 5.76 (1H, dq, J=6.9, 13.7 Hz, H-2´); 4.09 (2H, s, H-9); 3.66 (3H, s, H-8); 3.63 (2H, s, H-7); 2.79-2.93 (4H, s, H-4 and H-6); 1.78 (3H, dd, J=1.3, 6.9 Hz, H-3´).
